# SAGA Complex Components and Acetate Repression in *Aspergillus nidulans*

**DOI:** 10.1534/g3.112.003913

**Published:** 2012-11-01

**Authors:** Paraskevi Georgakopoulos, Robin A. Lockington, Joan M. Kelly

**Affiliations:** School of Molecular and Biomedical Science, University of Adelaide, Adelaide, SA, 5006, Australia

**Keywords:** acetate repression, SAGA complex, carbon catabolite repression, *creA*, *creB*, *creC*

## Abstract

Alongside the well-established carbon catabolite repression by glucose and other sugars, acetate causes repression in *Aspergillus nidulans*. Mutations in *creA*, encoding the transcriptional repressor involved in glucose repression, also affect acetate repression, but mutations in *creB* or *creC*, encoding components of a deubiquitination system, do not. To understand the effects of acetate, we used a mutational screen that was similar to screens that uncovered mutations in *creA*, *creB*, and *creC*, except that glucose was replaced by acetate to identify mutations that were affected for repression by acetate but not by glucose. We uncovered mutations in *acdX*, homologous to the yeast SAGA component gene SPT8, which in growth tests showed derepression for acetate repression but not for glucose repression. We also made mutations in *sptC*, homologous to the yeast SAGA component gene *SPT3*, which showed a similar phenotype. We found that acetate repression is complex, and analysis of *facA* mutations (lacking acetyl CoA synthetase) indicates that acetate metabolism is required for repression of some systems (proline metabolism) but not for others (acetamide metabolism). Although plate tests indicated that *acdX*- and *sptC*-null mutations led to derepressed alcohol dehydrogenase activity, reverse-transcription quantitative real-time polymerase chain reaction showed no derepression of *alcA* or *aldA* but rather elevated induced levels. Our results indicate that acetate repression is due to repression via CreA together with metabolic changes rather than due to an independent regulatory control mechanism.

Glucose metabolism can provide a primary source of energy and metabolic intermediates for eukaryotic microorganisms, and it is a preferred energy source. Carbon catabolite repression (CCR) is a global regulatory mechanism in microbial fungi that regulates the genes that are required for the use of alternative carbon sources, which are not transcribed highly when a source of repression is available (Katz and Kelly 2010). In *Aspergillus nidulans*, glucose, xylose, and sucrose are strong sources of repression, mannose, sorbitol, maltose, fructose, mannitol, and galactose lead to intermediate levels of repression, whereas arabinose, glycerol, melibiose, and lactose cause minimal repression (Arst and Cove 1973).

The molecular basis of glucose sensing and CCR in the single celled ascomycete, *Saccharomyces cerevisiae*, involves the Mig1p repressor protein which, signaled via the Snf1p kinase, acts together with the Ssn6p/Tup1p co-repressor complex to repress genes subject to CCR (Gancedo 1998; Turcotte *et al.* 2010). Studies of multicellular filamentous fungi indicate that although a Mig1p-like repressor protein, CreA, is involved, the mechanism differs considerably in filamentous fungi compared with yeast, and indeed there is variation within the filamentous fungi. The Tup1p and Snf1p homologs are not centrally involved in CCR in those multicellular filamentous fungi in which they have been studied (Hicks *et al.* 2001; Cziferszky *et al.* 2003), and at least in *Aspergillus nidulans*, the *creB* and *creC* genes may play a role. The *creB* and *creC* genes were identified in a mutation screen for genes showing derepression for the *amdS* gene (encoding acetamidase), and the mutant strains show pleiotropic derepression for a number of systems (Hynes and Kelly 1977). CreB is a deubiquitinating enzyme, and CreC is a WD40-containing protein present in a complex with CreB (Todd *et al.* 2000; Lockington and Kelly 2001).

Although *creA*-mediated CCR in *A. nidulans* has largely been studied with respect to glucose as the repressing carbon source, phenotype tests indicated that acetate can also lead to strong repression. Romano and Kornberg have shown that acetate, or a product of acetate metabolism, affects the uptake of some sugars, including glucose (Romano and Kornberg 1968, 1969); however, our evidence suggests that acetate also represses the expression of other pathways. Because acetate is metabolized via the glyoxylate bypass and gluconeogenesis, it was unexpected that repression by acetate would be as tight as repression by glucose for some systems.

In *S. cerevisiae*, the multisubunit *S. cerevisiae* SAGA (Spt-Ada-Gcn5-acetyltransferase) complex is required to activate transcription of a subset of RNA polymerase II−dependent genes (Baker and Grant, 2007; Rodriguez-Navarro 2009). Various components of the complex interact with the TATA-binding protein, mediate nucleosomal HAT activity, help recruit the basal transcription machinery, and target DNA-bound activators for recruitment to promoters. Genome-wide expression analysis indicates that although TFIID and SAGA share many subunits, they are responsible for the expression of different genes, with SAGA regulating genes that respond to environmental stresses (Baker and Grant 2007). SAGA performs multiple functions, including acetylating core histones, recruiting the preinitiation complex, and deubiquitinating histone H2B-Ub. In addition to its role in activating transcription, SAGA also promotes transcription elongation and export of the nascent mRNA (Samara and Wolberger 2011). The activities described for the yeast complex have been found to be conserved in Drosophila and human SAGA. Independent groups have demonstrated a differential requirement of the SAGA complex components Spt8p and Spt3p for efficient recruitment of TBP to promoters *in vivo* (Grant *et al.* 1998; Sterner *et al.* 1999, Bhaumik and Green 2002; Sermwittayawong and Tan 2006). Spt8p is required at only a subset of SAGA-dependent promoters, revealing a complex combinatorial network for transcription activation *in vivo* (Eisenmann *et al.* 1994; Bhaumik and Green 2002). Here, we present an analysis of mutations in SAGA components that affect phenotypic repression by acetate in *Aspergillus nidulans*.

## Materials and Methods

### Strains and media

The genotypes of *A. nidulans* strains are shown in Table 1. *Aspergillus* complete and minimal media are based on those described (Cove 1966). Carbon and nitrogen sources were added aseptically to the media to the final concentrations shown for each test. Transformation of *A. nidulans* was based on the procedure of Tilburn *et al.* (Tilburn *et al.* 1983).

**Table 1:** Genotype of *A. nidulans* strains

*Pseudonym*	Genotype	Derivation
Wild type	*yA1*; *riboB2*	Hynes (H17A12)
*nkuA*Δ	*pyroA4 [nku*::*argB]*; *riboB2*	Nayak *et al.* 2006
*acdX*Δ;*nkuA*Δ	*[acdX*::*A. f. riboB]*; *pyroA4 [nkuA*::*argB];riboB2*	This work
*^MYC^acdX;nkuA*Δ	*[A. f. riboB*::*^MYC^acdX]*; *pyroA4 [nkuA*::*argB]*; *riboB2*	This work
*sptC*Δ;^MYC^*acdX;nkuA*Δ	*[^MYC^acdX]*; *pyroA4[nkuA*::*argB]*; *[sptC*::*A. f. riboB] riboB2*	This work
*pyrG89*	*pyrG89;areA217;facA700*	This work
*areA217*	*yA1*; *areA217*; *riboB2*	(Hynes and Kelly 1977)
*areA217;facA700*	*pabaA4;areA217*; *facA700*	Lockington, unpublished data
*areA217;fac700;pyrG89*	*pabaA4 pyrG89;areA217*; *facA700*	This work
*ACDX*	*yA1*; *acdX1 areA217*; *facA700*; *pabaA4*	This work
*ACDX;facA+*	*yA1*; *acdX1 areA217*; *riboB2*	This work
*ACDX-2*	*yA1 pyrG89*; *acdX2 areA217*; *facA700;*	This work
*ACDX-2;facA+*	*yA1 pyrG89*; *acdX2 areA217*; *riboB2*	This work
*acdX*Δ*^a^*	*yA1;[acdX*::*A. f. riboB]*	This work
*acdX*Δ; *areA217**^a^*	*yA1;[acdX*::*A. f. riboB] areA217*	This work
*acdX*Δ; *areA217;creB1937**^a^*	*yA1 pabaA4*; *cre*B1937; *[acdX*::*A. f. riboB] areA217*	This work
*acdX*Δ; *creB1937**^a^*	*yA1*; *cr*e*B1937*; *[acdX*::*A. f. riboB]*	This work
*sptC*Δ*^a^*	*yA1 pabaA4*; *[^MYC^acdX]*; *[sptC*::*A. f. riboB]*	This work
*sptC*Δ; *areA217**^a^*	*yA1*; *areA217;[sptC*::*A. f. riboB]*	This work
*sptC*Δ; *areA217;creB1937**^a^*	*yA1*; *creB1937*; *areA217*; *[sptC*::*A. f. riboB]*	This work
*sptC*Δ; *creB1937**^a^*	*yA1 pabaA4*; *creB1937*; *[^MYC^acdX]*; *[sptC*::*A. f. riboB]*	This work
*creB1937*	*yA1 pabaA4;creB1937*	(Lockington and Kelly 2001)
*creB1937;acdX1*	*yA1*; *acdX1;creB1937*; *riboB2*	This work
*creB1937;acdX2*	*yA1*; *acdX2*; *creB1937*; *riboB2*	This work
*creC956;acdX1*	*yA1 acdX1*; *creC956 riboB2*	This work
*creC956;acdX2*	*yA1 acdX2*; *creC956 riboB2*	This work
*creA*Δ*4;areA217*	*yA1 creAΔ4;areA217*; *riboB2*	(Shroff *et al* 1997)
*creA204;areA217*	*yA1 creA204;areA217*; *riboB2*	(Hynes and Kelly 1977)
*creA218;areA217*	*yA1 creA218;areA217*; *riboB2*	(Hynes and Kelly 1977)
*creA221;areA217*	*yA1 creA221;areA217*; *riboB2*	(Hynes and Kelly 1977)
*creA331;areA217*	*yA1 creA331;areA217*; *riboB2*	(Shroff *et al* 1997)
*creB1937;areA217*	*yA1 creB1937;areA217*; *riboB2*	(Lockington and Kelly 2001)
*creC956;areA217*	*yA1 creC956;areA217*; *riboB2*	(Todd *et al.* 2000)

a Note the genotype status for the *riboB* locus has not been identified.

### Genetic screens

In the first screen, mutations were selected, in an *areA217*-containing strain, as suppressors of the phenotypic effects of *areA217* on 50 mM acetate and 10 mM proline medium. However, this screen uncovered mutations in the gene encoding acetyl CoA synthetise, *facA*.

To avoid isolating *facA* mutations, a second screen was carried out using an *areA217;facA700* double-mutant strain, which selected for mutations that cause loss of acetate repression of the *amdS* gene on 1% glycerol, 50 mM acetate, and 10 mM acetamide medium. The *facA700* allele was outcrossed from the mutant strain before analysis.

### Construction of *acdX* and *sptC* deletion strains

The *acdX* gene was deleted by homologous recombination using a linearized construct containing the *A. fumigatus riboB^+^* gene, flanked by sequences from the 5′ untranslated region and 3′ sequence toward the end of the gene, both derived by PCR of genomic DNA. The construct was transformed into a riboflavin requiring nonhomologous end joining deficient strain, which only permits homologous recombination (Nayak *et al.* 2006), to produce riboflavin independent transformants. The precise deletion that had occurred was confirmed by Southern transfer analysis of genomic DNA from representative transformants. The deletion begins at the *Bgl*II site upstream of the start codon and ends at the *Bgl*II site upstream of the stop codon, resulting in a deletion of 1006 bp. The *sptC* gene was deleted using an essentially identical construction, which was transformed into a strain that contained an *acdX* deletion and a MYC-tagged version of *acdX*. For *sptC* the deletion begins at the *Not*I site upstream the start codon and ends at the *Bgl*II site upstream the stop codon, resulting in a deletion of 1714 bp. Meiotic crosses were used to derive *acdX* and *sptC* deletions in the various genetic backgrounds used.

### RNA isolation

Conidia were used to inoculate liquid cultures that were incubated shaken at 37**°**C. Mycelia were grown on 3% lactose as sole carbon source [noninduced growth conditions (NI)]. After 18 hr of growth, the gratuitous inducer of *alcA* and *aldA*, ethyl methyl ketone (EMK), was added (50 mM; induced growth conditions, I). For repressing conditions, 1% glucose or 50 mM sodium acetate pH 6.0 was added simultaneously with EMK (induced repressed growth conditions, IG or IA). Cells were harvested after a further 4 hr. Then, 100 mg of wet weight of mycelia was used to extract total RNA using the RNeasy Plant Mini Kit following the manufacturer’s instructions (QIAGEN).

### Reverse-transcription quantitative real-time PCR (RT-qPCR)

Total RNA was treated using RNase-free DNase, and first-strand synthesis of cDNA was performed following the manufacturer’s instructions (Promega). RT**-**qPCRs were performed using an ABI Prism 7000 Sequence Detection System (Applied Biosystems) and *Power* SYBER Green PCR Master Mix (Applied Biosystems). The thermal cycling conditions used were as follows: an initial step at 50° for 2 min; 10 min at 95°; and 40 cycles of 15 sec at 95° and 1 min at 60°. In all experiments appropriate negative controls containing no template DNA, were subjected to the same procedure to exclude or detect any possible contamination. At least three independent growth and RNA extractions were performed for each strain and condition, and RT-qPCR was performed in triplicate for each sample. Cycle thresholds for each triplicate were averaged and normalized against the expression of *tubC* (β-tubulin), which was used as an endogenous control, as in Semighini *et al.* (Semighini *et al.* 2002). The relative standard curve method for quantification was used to determine the relative fold change in the expression of the experimental samples compared to the endogenous control. Table 2 lists the gene-specific primers used for amplification of the target cDNA and the cDNA from the ubiquitously expressed *tubC*. Primers were designed such that one of the primers from each pair spanned an exon-exon junction to eliminate amplification, and hence detection, of contaminating genomic DNA. The statistical significance of differences between experimental samples and controls was calculated using a two-tailed *t* test.

**Table 2:** Gene-specific primer pairs used for RT-qPCR

Gene Name	Forward Primer	Reverse Primer
*tubC*	*5′ AGG GCT TCC AGG TGA CGC 3′*	*5′ ACA CTT TCG GCG ACG GC 3′*
*alcA*	*5′ GAG GCT CTG GAC TTC TTC GCT 3′*	*5′ GCG ATT CTG CCT TGT TCC ATA 3′*
*aldA*	*5′ CGT CAC TAT CCA GAA GTT CAA GG 3′*	*5′ GGC AGC AGC AAG ACC GTA G 3′*
*alcB*	*5′ CAG TGC CCT CCA TGC CAG 3′*	*5′ GCA GGA CCG AGC ACG TAT TG 3′*
*alcC*	*5′ CGG CGT TGA GGC GTT AGA C 3′*	*5′ CAA TCT TCC CTT GCC CCA TC 3′*

RT-qPCR, reverse-transcription quantitative real-time polymerase chain reaction.

## Results

### Repression by acetate and glucose both require CreA but are separate

**Figure 1:**
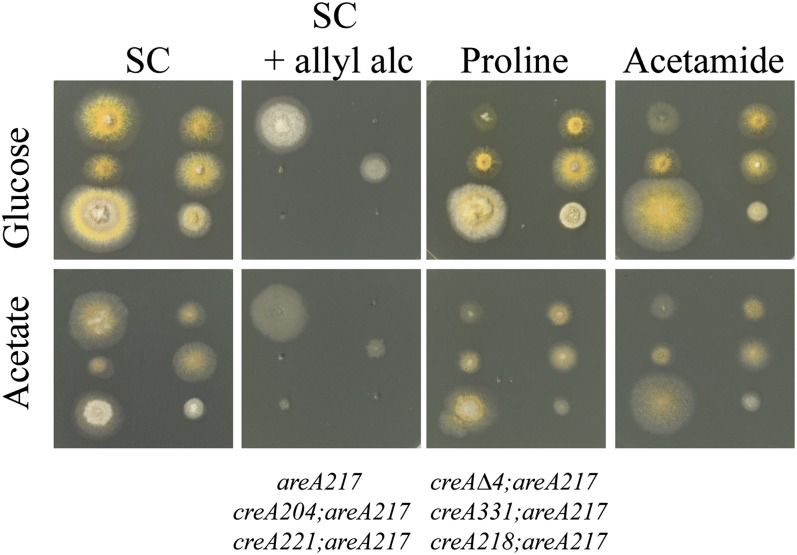
Mutations in *creA* affect repression by glucose and by acetate. Strains of genotype, indicated at the bottom of the figure, were grown on either 1% glucose (top row) media, or 50 mM acetate (bottom row) or containing 10 mM ammonium (SC), 10 mM allyl alcohol and 10 mM ammonium (SC + allyl alc), 10 mM proline (Proline), or 10 mM acetamide (Acetamide). *areA217* is used as the control strain to allow suppression of *areA217* to be scored on acetamide and proline media. Plates were incubated at 37° for 60 hr. Here, *areA217* is used as the control strain to allow the suppression phenotype to be assessed.

The majority of characterized *creA* mutations, including a precise deletion of the *creA* gene, abolish both acetate and glucose mediated repression when assayed on 1% glucose or 50 mM acetate media in standard tests for loss of carbon repression for genes subject to carbon repression. One such test is sensitivity to the presence of allyl alcohol in acetate or glucose media, which monitors expression of alcohol dehydrogenase (Dowzer and Kelly 1991). Another test is suppression of the effects of a loss of function *areA* mutation on acetate or glucose media for growth using proline or acetamide as nitrogen sources, as *are*A mutant strains can only use these compounds if carbon source repression of the genes required is relieved (Arst and Cove 1973). These tests demonstrate that both acetate mediated repression and glucose repression require a functional *creA* gene (Figure 1).

**Figure 2:**
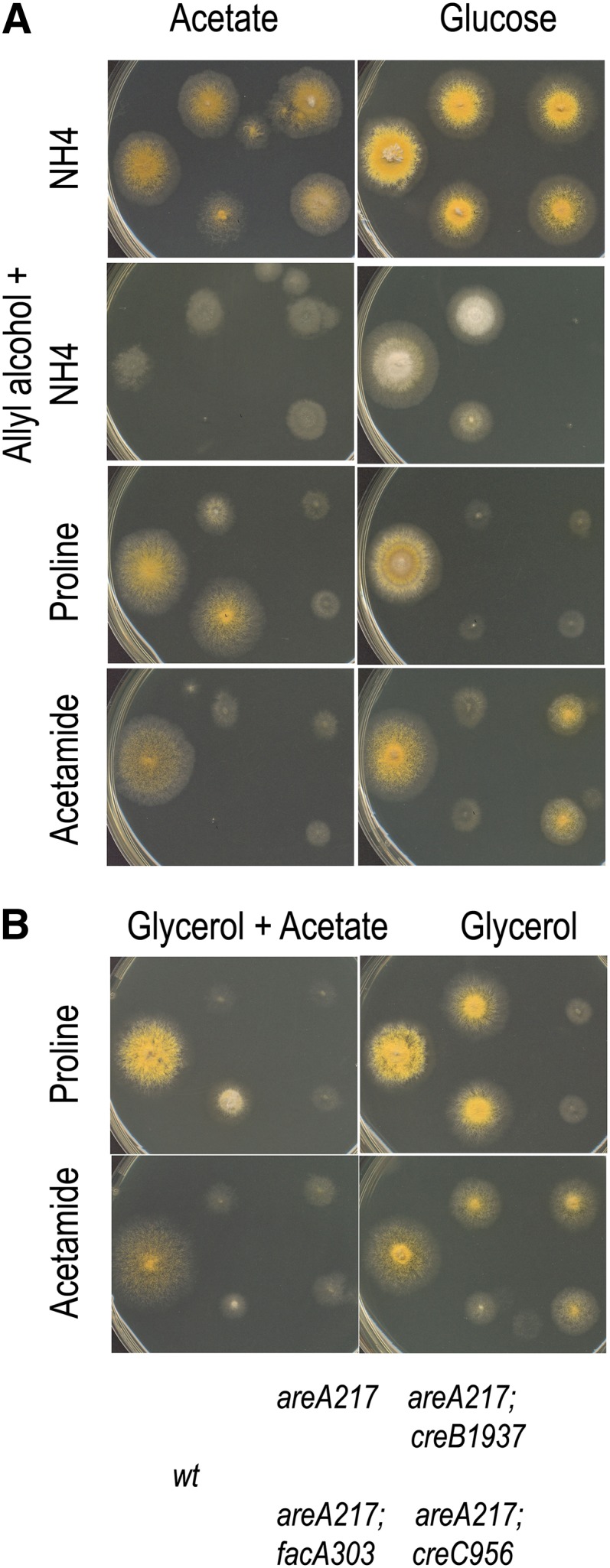
Mutations in *creB* and *creC* affect repression by glucose but not by acetate. Strains have the genotype shown in the key at the bottom of the figure. (A) Strains were grown on either 50 mM acetate (left column) or 1% glucose (right column) media, containing 10 mM ammonium (top row), 10 mM allyl alcohol and 10 mM ammonium (second row), 10 mM proline (third row), or 10 mM acetamide (fourth row). (B) Strains were grown on either 1% glycerol plus 50 mM acetate (left column) or 1% glycerol (right column) media, containing 10 mM proline (top row) or 10 mM acetamide (second row). Plates were incubated at 37° for 2 d.

The *creB* product, a deubiquinating enzyme (Lockington and Kelly 2001), and the *creC* product, a WD40 repeat protein (Todd *et al.* 2000), act together in a large complex (Lockington and Kelly 2002) and both are required for complete *creA*-mediated glucose repression. Loss-of-function mutants in either *creB* or *creC* lead to sensitivity to allyl alcohol in glucose medium, and suppression of the effects of the *areA217* mutation on glucose medium containing acetamide as nitrogen source but not on acetate media (Figure 2). The *creB* and *creC* mutations cannot be assessed for suppression of the effects of the *areA217* mutation on acetate or glucose media for use of proline due to their effects on proline uptake (Hynes and Kelly 1977).

Thus in these tests, the effects of glucose and acetate are both mediated via CreA, but the effects due to acetate are independent of the CreB/CreC complex. The fact that *creB* and *creC* mutants only affect repression by glucose implies that repression by glucose and acetate may involve independent signaling pathways that converge on the CreA repressor protein. That this might be the case is supported by the different metabolic requirements for growth on acetate (requiring gluconeogenesis) compared with growth on glucose, which in itself would make a common pathway for repression by both compounds unlikely. However, before this report, no mutations have been described that only affect the effect of acetate.

### Isolation of mutations affecting acetate repression in *A. nidulans*

Given the possibility that a separate pathway for acetate repression may exist, we performed screens similar to those used to select *creA*, *creB*, and *creC* mutations (Arst and Cove 1973; Hynes and Kelly 1977), using repressing levels (50mM) of acetate.

In the initial screen carried out in an *areA217* genetic background (see *Materials and Methods*) mutations in the *facA* gene encoding acetyl coA synthase were obtained, which prevented the use of acetate as a carbon source, and allowed proline to be used as a carbon and nitrogen source in the presence of acetate in the *areA217* background. However, *facA* mutations, including *facA303*, did not relieve acetate repression of the *amdS* gene encoding acetamidase, required for acetamide use when an alternative nonrepressing carbon source such as glycerol was provided to allow growth. This result shows that acetate repression of the proline pathway genes, but not the *amdS* gene, requires acetate metabolism, indicating the possibility of several paths to acetate repression.

**Figure 3:**
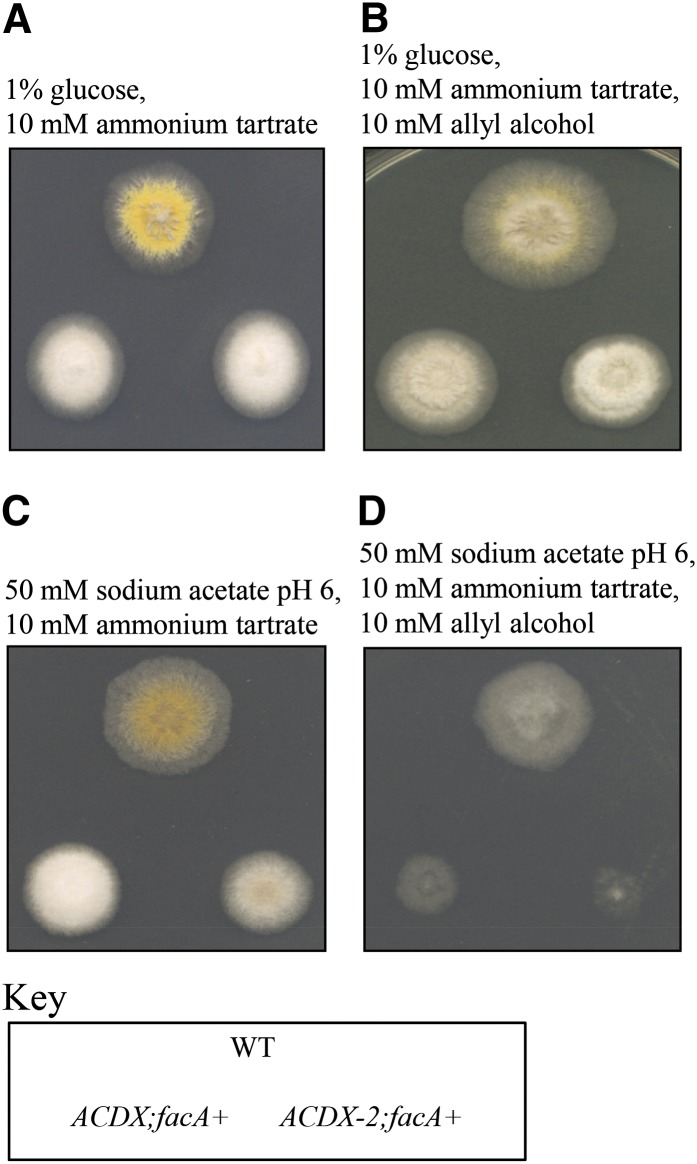
Initial phenotypic analysis of *acdX1 and acdX2* mutants. Strains with the genotype shown in the key at the bottom of the figure were grown at 37° on media as indicated above the panels for 3 d.

To avoid obtaining mutations in *facA*, a second screen was carried out using an *areA217;facA700* double mutant strain on 1% glycerol, 50 mM acetate, 10 mM acetamide medium selecting for loss of acetate repression of the *amdS* gene (see *Materials and Methods*). In this screen, a mutant strain was identified that had compact colony morphology with pale-colored conidia. This strain, ACDX1, grew better on glycerol plus acetate medium than the parent when proline or acetamide was present. The strain was out-crossed to remove the *facA700* mutation so that the acetate repression phenotype could be more directly monitored in the absence of the glycerol needed to provide a carbon source in a *facA700* strain. The *areA217;facA^+^* derivative of the ACDX1 strain showed stronger growth on acetate and proline medium than a strain containing *areA217* alone, indicating that acetate repression of proline utilization was reduced by the lesion in ACDX1, although the *facA^+^* derivative showed minimal phenotypic effect on acetate and acetamide medium, consistent with the earlier observation that acetate repression of proline and acetamide genes are different. However, there was no suppression of *areA217* when glucose was the carbon source, indicating that glucose repression was unaffected. No suppression of the *areA217* mutation was seen using nitrogen sources such as urate or nitrate, which require AreA protein but are not subject to *creA* mediated repression. The *areA^+^;facA^+^* derivative strain of ACDX1 was sensitive to allyl alcohol on acetate medium (Figure 3D), but no sensitivity to allyl alcohol was observed when repressing levels of glucose were present (Figure 3C).

To obtain more mutants involving acetate repression, a third screen was carried out using a *pyrG89;areA217;facA700* strain and the same selection process. A strain was selected, ACDX2, which strongly resembled the phenotype of the original ACDX1 strain.

### Molecular characterization of lesions in the ACDX1 and ACDX2 strains

The ACDX1 strain was crossed into a *pyrG89*-mutant background and the resulting strain was transformed with the *A. nidulans* replicating plasmid libraries (Osherov *et al.* 2000; Osherov and May 2000) selecting for the ability to grow without uridine and uracil, which selects for transformants that have the wild-type *pyrG* gene present on the vector plasmid. Transformants showing normal conidiation and growth habit were purified, and complementing plasmids were then rescued from the transformed strains’ DNA by electroporation into *Esherichia coli*.

A single plasmid was rescued from a fully complemented transformant of the ACDX1 mutant strain, and when transformed back into the *pyrG89* derivative of the ACDX1 mutant strain it was found to be capable of complete complementation of the mutant phenotype. The *A. nidulans* DNA insert was found to be chimeric, but through transforming the *pyrG89* derivative of the ACDX1 mutant strain with subcloned fragments the complementing region was shown to be present on a 3.5-kb *Kpn*I fragment of DNA. The only gene present on this region of the *A. nidulans* genome is ANID_04670.1 (Linkage Group III), and we designate this gene *acdX*. The gene encodes an uncharacterized WD40 repeat containing protein showing 28% identity and 43% similarity to the Spt8p transcription factor of *Saccharomyces cerevisiae* (supporting information, Figure S1), and the similarity is greatest within the WD40 repeat regions of the protein. *acdX* has a 5′ and 3′ intron whose positions are confirmed by the sequences of the two EST’s that have been mapped to the gene. The transcription start point and the 3′ end of the gene are also located by these two ESTs. A comparison with the highly conserved equivalent gene sequence from *Aspergillus oryzae* shows that the internal region between these two ESTs contains no other introns.

The ACDX2 mutant strain was also transformed with a plasmid containing the 3.5-kb DNA fragment containing the *acdX* gene, and this was found to completely complement the mutant phenotype. This mutation therefore is another mutant allele of *acdX*. The two mutations were designated *acdX1* and *acdX2*.

The *acdX1* and *acdX2* mutant genes were amplified by PCR from DNA isolated from the mutant strains and sequenced to determine the nature of the mutations. The *acdX1* mutation is a C to T change that creates a stop codon just beyond the final WD40 repeat, truncating the protein and abolishing a region that is highly conserved with Spt8p (Figure S1). The *acdX2* mutation is an insertion of an additional A in a sequence of 7 As causing a frame shift that is predicted to terminate the protein after two further amino acids. This mutation lies between the first three WD40 repeats and the last three WD40 repeats and, because only half of the protein product remains, probably results in a complete loss of function (Figure S1).

The phenotypes caused by the *acdX1* and *acdX2* alleles were similar, and to confirm that the phenotype conferred by *acdX2* represents the phenotype of a null allele, a strain in which *acdX* was deleted was constructed and analyzed. A construct was made in which the *Bgl*II fragment of *acdX* was replaced by the *A. fumigatus riboB* gene, and this construct was transformed as linear DNA into an *nkuA*Δ strain of *A. nidulans* (Nayak *et al.* 2006).

Riboflavin independent transformants were isolated, and all were phenotypically identical to the strain containing the *acdX2* allele, confirming that this represents the deletion phenotype. The *acdX*Δ mutation was crossed into an *areA217* mutant background and was found to be identical to the *acdX2* allele in strongly suppressing the effects of the *areA*217 mutation on proline utilization as nitrogen sources in the presence of acetate but not in the presence of glucose. Like the *acdX1* and *acdX2* alleles, *acdX*Δ also led to sensitivity to allyl alcohol in acetate medium when in an *areA217* mutant background with ammonium as the nitrogen source, or in a wild-type background when a derepressing nitrogen source such as nitrate is used.

### Genetic interactions with *creB* mutations?

The *creC* gene of *A. nidulans* encodes a WD40 repeat protein with some similarities to Spt8p and AcdX. The CreC protein has only five WD40 repeats, but in their spacing and type they resemble those present in Spt8p. As in Spt8p, there is a gap after the first repeat, followed by three closely packed repeats, and there is then a large gap until the next repeat. CreC, however, lacks the final two WD40 repeats that are present in Spt8p, but there is a region beyond the last repeat showing similarity to the conserved region beyond the final repeat of *acdX* that is lost in the *acdX1* mutation and thus may be important for function. Mutations in *creC* were obtained in similar screens using glucose media to those we have used to obtain *acdX* mutants using acetate media (Hynes and Kelly 1977). *creC* mutants are deficient in glucose mediated carbon repression but have no detectable affects on acetate-mediated carbon repression. Conversely, *acdX* mutants are deficient in acetate repression but have no detectable effects on glucose mediated carbon repression. Thus AcdX may play a similar role for growth on acetate media that CreC plays for glucose media. Both *creB* and *creC* mutations lead to resistance to high levels (33mM) of molybdate in glucose minimal medium (Arst 1981). Surprisingly, we have also found that all three *acdX* mutant alleles also lead to very strong molybdate resistance (data not shown). Although the molecular basis of the molybdate resistance is unknown, this again suggests that *acdX* and *creC* may be involved in similar processes.

**Figure 4:**
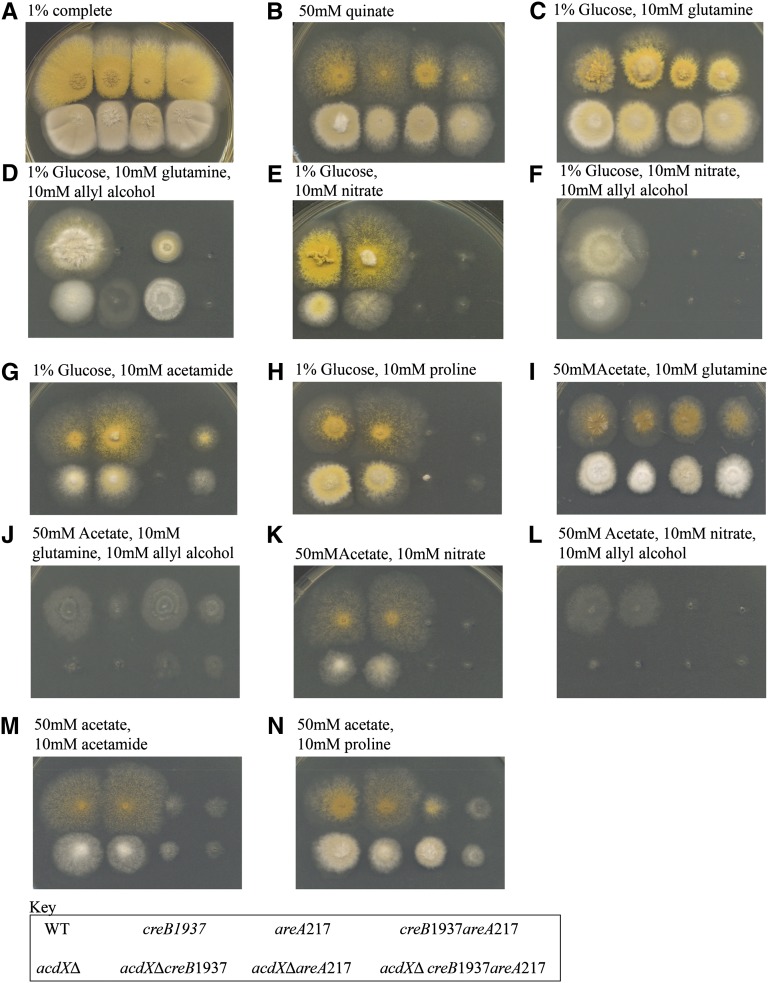
Phenotypic analysis of *acdX* mutants. Strains with the genotype shown in the key at the bottom of the figure were grown at 37° on media as indicated above the panels for 3 d.

Mutations in the *creB* gene were obtained in the same way as *creC* mutants and result in a similar phenotype to *creC* mutants. To investigate possible genetic interactions between AcdX and CreB, the *acdX*Δ allele was crossed into a *creB1937* mutant background. The *acdX*Δ progeny could easily be classified as wild-type or mutant for *creB* by testing for sensitivity to allyl alcohol in the presence of 1% glucose. The double-mutant strains were as sensitive to allyl alcohol on glucose media as the parent *creB* single mutant strain while retaining the *acdX*Δ phenotype of sporulation defects and defects in acetate repression (Figure 4, C *vs.* D and E *vs.* F).

The *creB* mutant strain was unable to properly use quinate as carbon source, or proline under either glucose repressing or derepressing conditions, because the *creB* gene product is required to stabilize the relevant permeases (N. Kamlangdee and J. M. Kelly, unpublished data). *acdX*Δ leads to marginally stronger growth on 50 mM quinate, although the morphological effects make this difficult to interpret (Figure 4B). The *acdX*Δ*;creB1937* double-mutant strain appears stronger than the *creB1937* strain but not as strong as the *acdX*Δ strain.

For the strains with a deletion of *acdX*, toxicity is observed when grown on media containing 50 mM proline. Proline toxicity has been previously reported, and considerable toxicity occurred as a result of mutations in the *prn* cluster (Arst *et al.* 1981). The toxicity seen on proline medium (Figure 4, H and N) implies that AcdX has a role in the regulation of some genes in the proline metabolic pathway, and therefore a deletion of *acdX* would lead to the accumulation of proline intermediates and consequent toxicity. Arst *et al.* (1980) demonstrated that the inability of *areA* mutants to use proline as a nitrogen source is due to insufficient uptake. In strains grown on medium containing 1% glucose and 10 mM proline there is little difference between the *acdX*Δ;*areA217* double-mutant strain and the *areA217* strain (Figure 4H); however, suppression of the *areA217* phenotype is observed in the presence of 50 mM acetate and 10 mM proline (Figure 4N). In the presence of acetate, *acdX*Δ may suppress the *areA217* mutation for proline use by bypassing the requirement of AreA, resulting in the derepression of *prnB*, which encodes the PrnB permease required for proline uptake. *acdX*Δ did not have an effect on the *areA217*-mutant phenotype when acetamide was used as the nitrogen source, further supporting that AcdX has a specific role for acetate repression of the proline metabolic pathway. These studies of *acdX*Δ*;creB1937* revealed no genetic interactions between AcdX and CreB.

### Deletion of the *A. nidulans* Spt3p homolog

In *S. cerevisae* mutations in the *SPT3* gene have almost identical consequences to *SPT8* mutations. Both gene products are involved in interactions with the TATAA binding protein and are subunits of the SAGA complex. Because of this, we investigated the effects of deleting the *A. nidulans* equivalent of Spt3. Searching the *A. nidulans* genome confirmed the existence of a well-conserved homolog of the yeast Spt3p, ANID_00719.1 (Linkage Group VIII), and we designated the gene encoding this protein *sptC*. The first intron is 460-bp long, which is unusual for an *A. nidulans* intron, and this was confirmed by analysis of the equivalent gene from *A. fumigatus*. A construct essentially identical to that used to delete the *acdX* gene was created using sequences flanking ANID_00719.1 and this was used to delete the *sptC* gene in the ^MYC^*acdX nkuAΔ* strain. Riboflavin-independent transformants were obtained, which appeared to be almost identical in morphology to *acdX* mutant strains. The *sptC*Δ strains were slightly more impaired in their growth rate, and colonies appeared to be more compact than *acdX* mutant strains.

**Figure 5:**
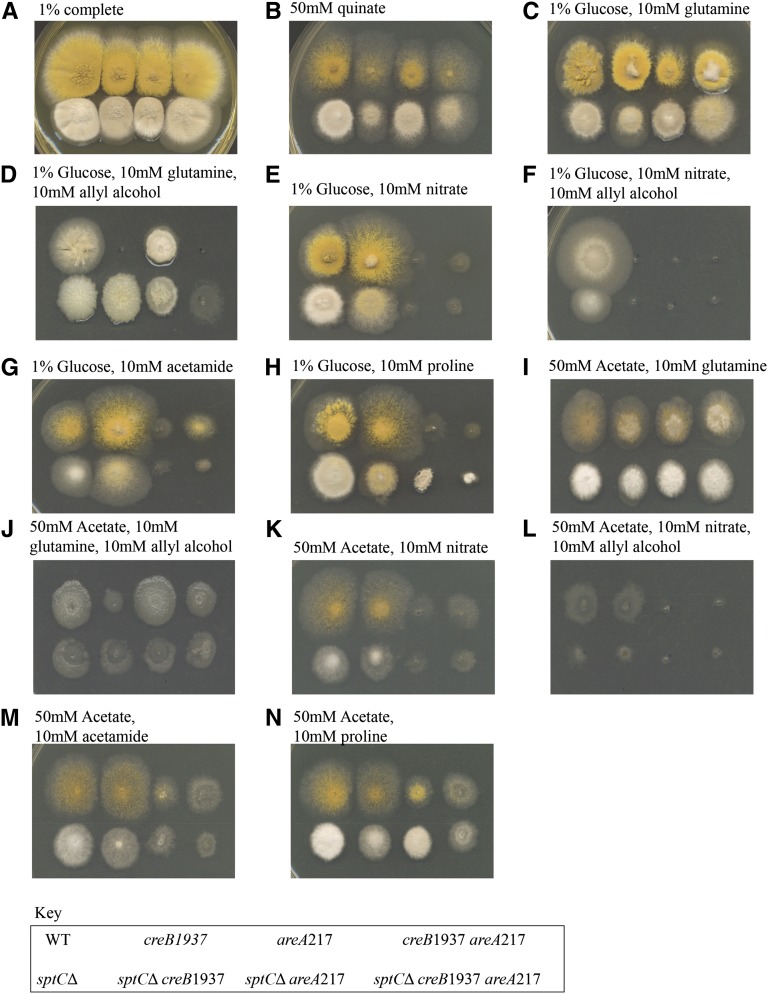
Phenotypic analysis of *sptC* mutants. Strains with the genotype shown in the key at the bottom of the figure were grown at 37° on media as indicated above the panels for 3 d.

A *sptC*Δ strain was crossed into an *acdX*^+^ *areA217* mutant background, and the effects of the deletion on suppression of *areA217* for use of various nitrogen sources in the presence of acetate or glucose as carbon sources were investigated. The *sptC*Δ strain showed very similar phenotypes to the *acdX*Δ strain in most genetic backgrounds and growth conditions tested (compare Figure 4 and Figure 5). As for the *acdX*Δ, no sensitivity to allyl alcohol was observed in the presence of 1% glucose, and *sptC*Δ strains were sensitive to 10 mM allyl alcohol in the presence of 50 mM acetate but not as sensitive as the *acdX*Δ strains (Figure 5, C *vs.* D and E *vs.* F).

*sptC*Δ was crossed into a *creB1937*-mutant background and the phenotypes were similar to those seen in the doubly mutant strain with *acdX*Δ (compare Figure 4 and Figure 5), which was not surprising because in *S. cerevisiae* their functions have been shown to overlap. However, there were some differences, indicating that SptC also plays a role in the presence of glucose. *sptC*Δ appears to suppress the *creB1937* phenotype on 1% glucose and 10 mM allyl alcohol when 10 mM glutamine is used as a nitrogen source, but not on any other nitrogen source tested (Figure 5, C *vs.* D and E *vs.* F). Thus, the *creB*1937 strain is sensitive to 10 mM allyl alcohol in the presence of 50 mM acetate when glutamine is used as the nitrogen source but not on any other nitrogen source tested. These results are specific to the nitrogen source used, *i.e.*, glutamine, indicating that SptC and CreB have a role in the regulation of the glutamine metabolic pathway; however, further experiments are required to elucidate the molecular basis of these results. Suppression of the *areA217* phenotype in the presence of 50 mM acetate and 10 mM proline was observed, and there was also suppression on 1% glucose and 10 mM proline that led to toxicity, indicating that not all genes in the pathway were equally affected (Figure 5H). Again, there was no suppression of the *areA217* phenotype in the presence of 10 mM acetamide (Figure 5G).

### Effects of deletion of *acdX* and *sptC* on expression of the *alc* and *ald* genes in *A. nidulans*

One line of evidence that *acdX* affects acetate repression is the sensitivity to the presence of allyl alcohol in acetate medium, indicating transcriptional derepression of an alcohol dehydrogenase (Dowzer and Kelly 1991). Because there are a number of alcohol dehydrogenases in *A. nidulans*, to allow interpretations to be drawn from this phenotypic test, relative transcription levels for *alcA* encoding alcohol dehydrogenase I, the enzyme required for ethanol metabolism (Lockington *et al.* 1985), *alcB* encoding alcohol dehydrogenase II, induced during carbon starvation (Hunter *et al.* 1996; Jones *et al.* 2001), *alcC* encoding alcohol dehydrogenase III, the enzyme involved in anaerobic survival (Sealy-Lewis and Lockington 1984; McKnight *et al.* 1985; Kelly *et al.* 1990), and *aldA* encoding aldehyde dehydrogenase, the enzyme required for acetaldehyde metabolism (Pickett *et al.* 1987), were determined using RT-qPCR.

Three strains were used for these experiments: *areA217*, which for the purpose of these experiments was used as the wild-type control; and the two experimental strains, *acdX*Δ*areA217* and *sptC*Δ*areA217*. The strains were grown under different physiological conditions: non-induced (NI); induced by the gratuitous inducer EMK (I); glucose repressed in the presence of EMK (IG); and acetate repressed in the presence of EMK (IA). The designation of conditions as induced or repressed refers to effects on *alcA* and *aldA*. In addition to the aforementioned physiological conditions, for the transcriptional analysis of *alcB*, the strains were also subjected to carbon-starved conditions (S).

**Figure 6:**
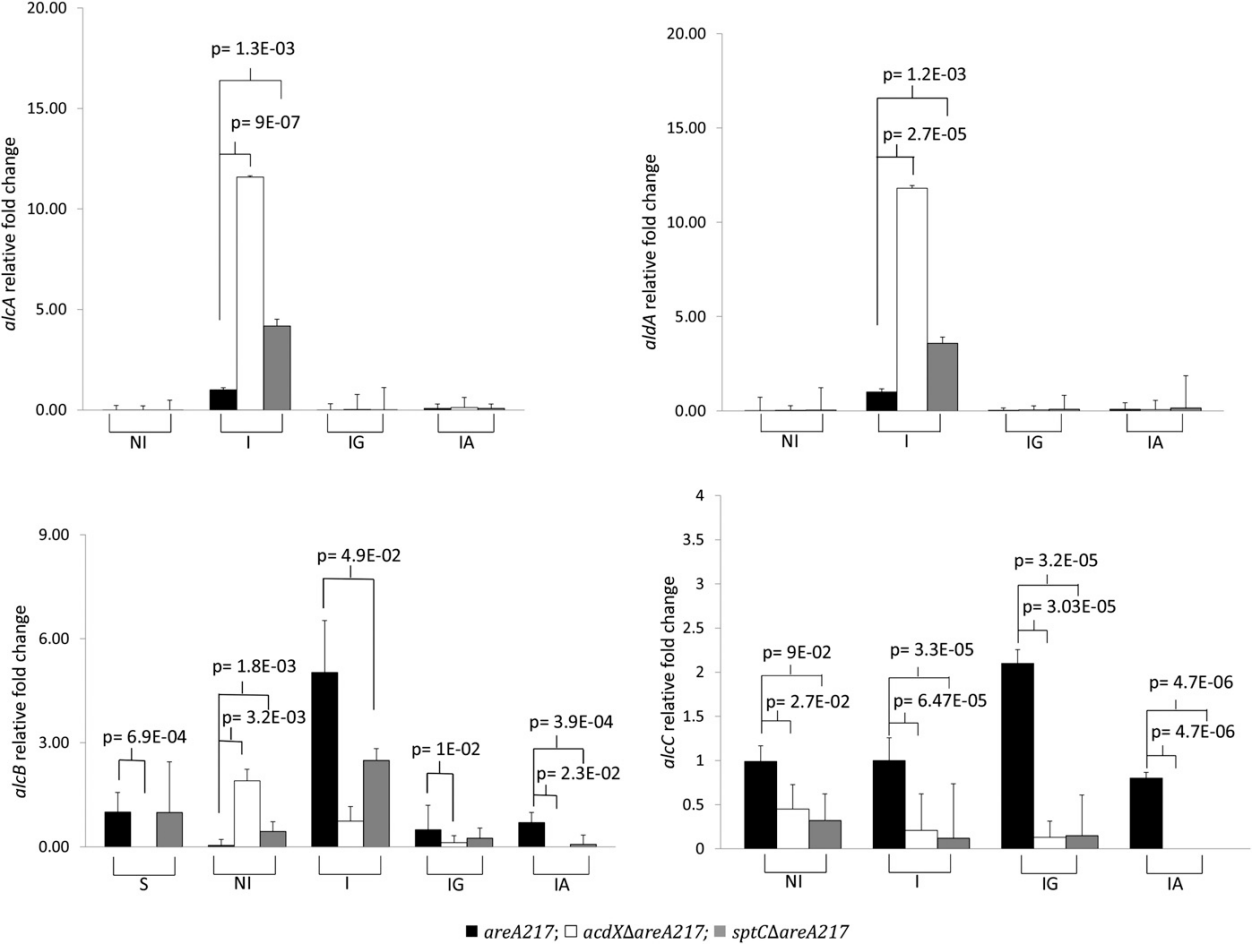
Relative quantitation of *alcA*, *aldA*, *alcB*, and *alcC*. The strains (*areA217*; *acdXΔareA217*; *sptCΔareA217*) were grown for 18 hr in 3% lactose, when the source of induction or repression was added and the strains incubated for a further 4 hr under the following physiological conditions: noninduced (NI), induced by the gratuitous inducer of *alcA* and *aldA*, EMK, (I). Induced with EMK, repressed with glucose (IG) and induced with EMK, repressed with acetate (IA); for the analysis of *alcB* the strain were grown under carbon-starved conditions. Cycle thresholds for each triplicate were averaged and normalized against the expression of *tubC*. Fold changes in gene expression are shown relative to I *areA217* for *alcA*, *aldA* and *alcC*; S *areA217* for *alcB*. The results are a representative of three repetitions. Significance values are for comparisons to the *areA217* strain within each physiological condition (two-tailed *T*-tests). Error bars represent the standard errors of the means.

For both *alcA* and *aldA*, RT-qPCR showed that deletion of *acdX* or *sptC* resulted in elevated induction, but there was no detectable level of derepression in mycelia grown in acetate or glucose cultures (Figure 6). The results obtained from the RT-qPCR indicate that the loss of neither AcdX nor SptC relieves acetate repression of these genes; thus, reinterpretation of the phenotypic tests was required.

For *alcB*, RT-qPCR showed that deletion of *acdX* or *sptC* resulted in elevated levels in conditions that were noninducing for *alcA* (NI), but levels were generally equal or lower than those of the wild type in all other conditions tested. This result also indicates that *alcB* is not required for acetate repression because no significant derepression was observed. This result was not unexpected because it has been previously shown that *alcB* is induced during carbon starvation (Hunter *et al.* 1996; Jones *et al.* 2001), and Huisinga and Pugh (2004) reported that the SAGA complex of *S. cerevisiae* is involved in the up-regulation of genes in response to environmental stresses including carbon starvation. In addition to addressing whether the phenotype observed in solid medium containing acetate and allyl alcohol was due to the transcriptional regulation of *alcB*, this RT-qPCR, specifically the S conditions of this experiment, was also able to address whether the SAGA complex components AcdX and SptC were required for *alcB* regulation in response to carbon starvation. The results show no significant indication that AcdX or SptC are required for the transcriptional regulation of *alcB* in carbon starved mycelia.

For *alcC*, RT-qPCR showed variable results, but there was no evidence that deletion of *acdX* or *sptC* resulted in increased levels in media containing either glucose or acetate. This result was expected because it has previously shown that *alcC* is not subject to CCR, and unlike *alcA* and *aldA* the regulation of *alcC*, is mainly at the posttranscriptional level (Kelly *et al.* 1990).

### Deletion of genes encoding two *A. nidulans* deubiquitinating enzymes

The phenotypes due to the *acdX* mutations on acetate media are similar to the phenotypes due to *creC* mutations on glucose media, and the overall structures of Spt8p, AcdX, and CreC are similar. Thus, we determined whether two deubiquitinating proteins present in the *A. nidulans* genome were partners for AcdX similar to the CreB deubiquitinating partner for CreC. The deubiquitinating enzyme Ubp8p is a component of yeast SAGA that is required for deubiquitination of histone 2B (Henry *et al.* 2003) and optimal gene activation, and ANID_03711.1 (Linkage Group II) encodes the most similar protein in the *A. nidulans* genome. CreB has a long carboxyl terminal extension, and the only other DUB predicted to have a C-terminal extension is ANID_02267.1 (Linkage Group VII). A deletion of each of these genes was made in a *nkuA*Δ strain of *A. nidulans* (Nayak *et al.* 2006), and each strain was similar to the wild type for acetate repression. Thus, ANID_03711.1 and ANID_02267.1 are unlikely to be partner proteins for AcdX.

## Discussion

Initially, we used direct and indirect plate assays to indicate that both glucose and acetate led to repression. One test involved the use of allyl alcohol, which is toxic to *A. nidulans* if broken down to acrolein by alcohol dehydrogenase activity, thus inclusion of this compound in glucose or acetate medium is a sensitive assay of alcohol dehydrogenase activity. Another test takes advantage of the phenotype caused by *areA* mutations (Arst and Cove 1973). AreA is required for growth on a range of compounds that can provide nitrogen to the cell, and those that can provide both carbon and nitrogen are regulated by both carbon and nitrogen global repression mechanisms, with the relief of either one sufficient for expression. When glucose or acetate is present with these compounds, *areA* mutant strains fail to grow, indicating both are repressing carbon sources. Previous research has demonstrated that CCR by glucose requires the CreA protein (Dowzer and Kelly 1991), and we used the phenotype tests described previously to determine that CreA also was required for repression by acetate. The CreB-deubiquitinating enzyme is required for full repression by glucose but not by acetate. These results encouraged us to use the same selection technique that had been used to identify *creA* and *creB* mutations, but substituting glucose with acetate.

Immediately, we found that the acetate repression phenotype was complex. In the first experiment *facA* (acetyl CoA synthetase) mutations arose that were apparently derepressed for the acetamide pathway but not the proline pathway, indicating that acetate metabolism was required for the acetate repression phenotype for some systems. This was reminiscent of the findings of Romano and Kornberg (1969), who found that acetate inhibition of the uptake of glucose was abolished in a *facA* mutant strain, suggesting that acetyl CoA may be required (Romano and Kornberg 1969). Screens in a *facA* mutant background uncovered *acdX* mutations that led to sensitivity to allyl alcohol and suppression of the *areA* mutant phenotype, indicators of derepression. However, when we directly measured the levels of alcohol dehydrogenases and aldehyde dehydrogenase in mycelia grown in various conditions using RT-qPCR, no transcriptional derepression was evident that could account for the sensitivity observed on solid medium containing allyl alcohol and acetate as the sole carbon source. This is quite different to the effects of *creB* mutants on glucose repression, where direct RT-qPCR measurement of *alcA* and *aldA* expression in mycelia showed derepression (T. A. Morris and J. M. Kelly, unpublished data). It is possible that for *acdX*, these apparently conflicting results between plate growth tests and liquid cultures are due to a combination of the expression of the *alc* and *ald* genes in dormant conidia, the difference in expression in conidia *vs.* mycelia, and the growth rate on the carbon source used. In *A. fumigatus*, Lamarre *et al.* (2008) showed, in experiments undertaken to understand the molecular mechanisms underlying the conidial exit from dormancy, that dormancy is associated with fermentation and reduced aerobic metabolism. Transcriptomic analysis showed that within the first 30 min of incubation of conidia in a rich medium, genes coding for alcohol dehydrogenases were down-regulated, whereas genes that function in the TCA cycle were up-regulated (Lamarre *et al.* 2008). These results suggest a shift from fermentation in dormant conidia to respiration as the germination process commences. In addition, Teutschbein *et al.* (2010) showed significant activity of alcohol dehydrogenase in resting conidia of *A. fumigatus*, which is strongly increased in mycelia grown in ethanol, whereas low expression and no activity were detectable in mycelia grown in glucose (Teutschbein *et al.* 2010). If the situation is similar in *A. nidulans*, these observations could explain the apparent contradiction of the phenotypic tests and the RT-qPCRs. When plated on solid glucose media, conidia are able to germinate rapidly and efficiently since glucose is a rich and repressing carbon source, and even if there is alcohol dehydrogenase in resting conidia phenotypic sensitivity to allyl alcohol is not observed due to rapid repression. However, with acetate as the sole carbon source, some sensitivity is expected because acetate is a poor carbon source and therefore growth and repression are slower, such that pre-existing alcohol dehydrogenase is able to convert allyl alcohol to the toxic compound acrolein. This finding is supported by the partial sensitivity observed for the wild-type strain on solid acetate and allyl alcohol medium, compared with the resistance on solid glucose and allyl alcohol medium. Although deletion of *acdX* did not lead to derepression of *alcA* in liquid cultures, up to 12-fold elevation was detected in induced conditions (Figure 6). If the deletion of *acdX* also leads to elevation of *alcA* in resting conidia, this would result in a greater conversion of allyl alcohol to acrolein than in the wildtype strain, which would account for the degree of sensitivity observed. Further experiments are required to confirm this.

This analysis has shown that AcdX is not involved in a regulatory mechanism of acetate repression and further that there is not a single mechanism for repression by acetate. In fact, the apparent acetate repression might be accounted for by acetate metabolism via acetyl CoA and effects on uptake of compounds and intracellular levels of inducers.

Given the role of Spt8p and Spt3p in the SAGA complex, and SAGA’s role in the stress response, we wanted to understand AcdX and its role in the SAGA complex. A strain deleted for the *SPT3* equivalent, *sptC*, was similar to the *acdX* deletion strain in that it showed apparent effects on acetate repression using the phenotypic analysis, but there was no derepression of *alcA* or *aldA* in RT-qRCR experiments. Interestingly, phenotypic effects were observed for the use of glutamine, proline and quinate, and these compounds are also affected by *creB* and *creC* mutations.

These studies have shown that mutations in *acdX* and *sptC*, identified as affecting repression by acetate but not by glucose, do not show derepression at the transcriptional level, rather elevated induction. Further, we have shown that repression in acetate media is complex, and studies in strains lacking acetyl CoA synthetase indicate that acetate metabolism is required for repression of some systems such as proline metabolism, but not for others such as acetamide metabolism. Our results indicate that acetate repression is due to repression via CreA together with metabolic changes, rather than due to an independent regulatory control mechanism.

## Supplementary Material

Supporting Information
