# Supplementary material for: SAGA Complex Components and Acetate Repression in Aspergillus nidulans
Source: G3 (Bethesda). 2012 Nov 1;2(11):1357–67. doi: 10.1534/g3.112.003913 (PMC3484666; doi:10.1534/g3.112.003913)
Supplement: Supporting Information [file supp_2_11_1357__index.html]

Supporting Information 

# SAGA Complex Components and Acetate Repression in *Aspergillus nidulans*

## Supporting Information for Georgakopoulos, Lockington, and Kelly, 2012

**Files in this Data Supplement:**

- Figure S1 - The top panel shows the AcdX amino acid sequence aligned to Spt8, showing 28.4% identity (PDF, 106 KB)
