# Supplementary material for: SAGA Complex Components and Acetate Repression in Aspergillus nidulans
Source: G3 (Bethesda). 2012 Nov 1;2(11):1357–67. doi: 10.1534/g3.112.003913 (PMC3484666; doi:10.1534/g3.112.003913)
Supplement: Supporting Information [file supp_2.11.1357_FigureS1.pdf]

AnAcdX vs ScSpt8

```
                                                                                10                  20         30
                                                                M.........ASIVEDEDD............RDIAGSQDGSSDN.DMDDTLRDADEGGG
                                                                :          .:.::.:             .:. :..::. :. :  ::  .: :.
                                                                MDEVDDILINNQVVDDEEDDEEMLSGLENDSKQDLEGNDDGGEDEEDDDDDDEDDDDDED
                                                                         10        20        30        40        50        60
40          50                      60        70        80         90        100              110       120
DNEPD...MDADGDAD...............DQDADSASNASHASESAEVATQQNQETTMTPVPDNA..TTDLTSVFHP.SVRPECLT...........ASSYDIVPTTAAPHSTSINA
. : :    : ::. :               :..  .:..:. :..:....:. ..: .      :..  . . .:  :  :.  :              :.::.: ::.: : .: .::
EREDDDEQEDDDGEDDAARMDKTATPTNEHQHDEQKAAAAGAGGAGDSGDAVTKIGSEDVKLSDVDGGVGSREASSSTHEASANGEVYEYYKHMLNAAQIADSYNIYPTAAIPIQTHVNA
        70        80        90        100       110       120       130       140       150       160       170       180
   130       140       150       160       170       180            190                 200       210       220       3
ITATADMRWVFSGGSDGYVRKFNWVDSINSKLMLTVAQRHPFVDSVIKAGVLMTYWEN..........MDGNA......LSPVYSLACQSEGLWLLSGLESGSIRLQSIRHDEGKEIALL
....  ....: ::::::.::.. ......:: ::. :.: ...:. .::.:..::::          ...:        .:::.::  ::: :..::::..:.: .:..:. ::.     .
LAVSRGLKYLFLGGSDGYIRKYDLLNTLEGKLSLTILQKHSLAESIQNAGILQSYWENEIPQKKSEMKLSANKTDYEPKVSPVHSLEVQSECLFILSGLQNGGITMQGVRYMEGSIAHYF
       190       200       210       220       230       240       250       260       270       280       290       300
30          240       250       260       270       280        290        300         310       320       330       340
QQ...HTSAVSVLSLTSDEKSLLSGSWDKRIYDWDLNTGQTRRVFGSSAGQISAIELRP.ESSLPVPRDTTEIQQPNGTFSSNNQASGGNSFSYMDTTNDQGDNDAVNPQAGSPADSLFG
.    ::. :..: :..:  .::::::::. .:::.::.    : .: ...:..:.::  ::. :                 :.:  ::          :...:.:
KGRNGHTQIVNILRLNGQEDRFLSGSWDKRLLEWDLQTGDIVNEFKKSRSELSSLEMRPLYSSVDV...............SGNVNSG........KENENADDDM..............
       310       320       330       340       350       360                     370                380
   350       360       370       380       390       400       410       420       430       440       450       460
GADSLFGDADGTAGDGLGTATNSFGIDDDDEFGKALTNGVAPDADAAGEPDTVQQKNLFDSKDPSNDAPGVDSNTLVPNQPLDSHSTDAVNNQSQPLVNGLPHAEELEPPSQSQEHTQST
  :::::                   .:.::              ::..::       .. : :                                  ::  . .:..   : .  . .:.
..DSLFGD.................EDEDE...........KQDAGNEP........VETGDGS................................NGEENKEQISEESLNIVYDESV
                         390                    400                                                410       420       430
   470       480       490       500         510       520       530       540       550       560       570       580
PTEASNDNDNTFLAASIDGTIRVWDRRQPSAIARITPRNS..PPWCMNACWSPDGNYIYAGRRNGTVEEYSLHKGLREPERTFKFPQGSGPVTALKAMPNGRHLLCASHDILRLYDLK..
           :.....:....::::. .. :    :..  ::::..:::. ::...::::::. :::...: :    .: ...:.:. ::::. .::::::.:::::::.: .:::...
..........FMTSGLNGSVHIWDRRMTQSPALSLERGAGVPPWCLSACWGVDGDHVYAGRRNACVEQFDL.KMPSKPIHNLKLPSISGPVSCVKAMPNNKHLLCASRDNIRLYNVEIA
                440       450       460       470       480       490        500       510       520       530
        610       600       610       620       630       640
...HEQATRHSTVPFLIIPGHRTGTVSQLYVDQACRFLVSTSGNRGWEGSTTEVLLGYEIGVPVVR
    ...:. : ::::::::. : .:.::.: . ::..:::::::::.:.:.. : :.: .    .
VDASNSTTKSSKVPFLIVPGHHGGIISNLYLDPTSRFIISTSGNRGWQGNSTDTTLIYDIDL...E
        550       560       570       580       590       600
```

AnSptC vs ScSptC

```
        10        20        30        40        50         60        70        80        90        100          110
MSSDRTPKYRQEIQQMMFVSGETAEPSIETTTLIEDIVRQQVVELLARSTALATRRGVRSISTDDLIFLIRHDKAKVSRLKTFLSWKDVRKNVKDSDDKGGADAADF...AGADDPM..A
: . .  ::: ::::::::::  .: .:::.:::::: ::.:.: .:.   :   :: :::   .:.:::::::::::.::.:.::::::.:::.::.:  ..:. ..     ::..: .  :
MMDKH..KYRVEIQQMMFVSGEINDPPVETTSLIEDIVRGQVIEILLQSNKTAHLRGSRSILPEDVIFLIRHDKAKVNRLRTYLSWKDLRKNAKDQDASAGVASGTGNPGAGGEDDLKKA
          10        20        30        40        50        60        70        80        90       100       110
 120       130       140       150          160       170       180       190       200       210       220       230
GGVVAGPQDVASKPKNKKARVGLAWDVNSFYSVQVPERDDEED...EEEEEQNYATLQRLAADERTKHMTREEYVFWSECRQASFTYRKSKRFREWAGFGIVTESKPNDDIVDILGFLT
::     .: ..  : ::... : :... ...  : .:..:     :.:.: : .::.::  ::.::..::.:::: ::.::::::::.::.:::..:.:.. .::.::.::..:::::::
GGGEKDEKDGGNMMKVKKSQIKLPWELQFMFNEHPLENNDDNDDMDEDEREANIVTLKRLKMADDRTRNMTKEEYVHWSDCRQASFTFRKNKRFKDWSGISQLTEGKPHDDVIDILGFLT
120       130       140       150       160       170       180       190       200       210       220       230
     240       250       260             270       280       290       300       310        320       330
FEIVQTLTEEALKVKEREDREKNRRGGAENSAED.......TKKRKRETGLFDPPEEGRTPVEPRHIREAYRKLQATPNKNIAML.LHNGRLPARMPLRLFLMGLSI
:::: .::: :::.:.::.  ....   ...:..:         .. .:.   ::: ::.   .:..::::.::.: ::.    .. :.   ..::: .. :.    .:
FEIVCSLTETALKIKQREQVLQTQKDKSQQSSQDNTNFEFASSTLHRKKRLFDGPENVINPLKPRHIEEAWRVLQTIDMRHRALTNFKGGRLSSK.PI...IM
         250       260       270       280       290       300       310       320       330
```

Figure S1 The top panel shows the AcdX amino acid sequence aligned to Spt8, showing 28.4% identity. *acdX1* truncates after D indicated at amino acid 609, and *acdX2* truncates after K indicated at amino acid 400. The bottom panel shows SptC aligned to Spt3, showing 47.0% identity. Sequences were aligned using the MUSCLE alignment tool via Geneious Pro 4.7.5.
